# Multi-omics analysis defines core genomic alterations in pheochromocytomas and paragangliomas

**DOI:** 10.1038/ncomms7044

**Published:** 2015-01-27

**Authors:** Luis Jaime Castro-Vega, Eric Letouzé, Nelly Burnichon, Alexandre Buffet, Pierre-Hélie Disderot, Emmanuel Khalifa, Céline Loriot, Nabila Elarouci, Aurélie Morin, Mélanie Menara, Charlotte Lepoutre-Lussey, Cécile Badoual, Mathilde Sibony, Bertrand Dousset, Rossella Libé, Franck Zinzindohoue, Pierre François Plouin, Jérôme Bertherat, Laurence Amar, Aurélien de Reyniès, Judith Favier, Anne-Paule Gimenez-Roqueplo

**Affiliations:** 1 INSERM, UMR970, Paris-Cardiovascular Research Center, F-75015 Paris, France; 2 Université Paris Descartes, Sorbonne Paris Cité, Faculté de Médecine, F-75006 Paris, France; 3 Programme Cartes d’Identité des Tumeurs, Ligue Nationale Contre Le Cancer, 75013 Paris, France; 4 Department of Genetics, Assistance Publique-Hôpitaux de Paris, Hôpital Européen Georges Pompidou, F-75015 Paris, France; 5 Hypertension Unit, Assistance Publique-Hôpitaux de Paris, Hôpital Européen Georges Pompidou, F-75015 Paris, France; 6 Department of Pathology, Assistance Publique-Hôpitaux de Paris, Hôpital Européen Georges Pompidou, F-75015 Paris, France; 7 Department of Pathology, Assistance Publique-Hôpitaux de Paris, Hôpital Cochin, F-75006 Paris, France; 8 Department of Digestive and Endocrine Surgery, Assistance Publique-Hôpitaux de Paris, Hôpital Cochin, F-75006 Paris, France; 9 INSERM, U1016, Institut Cochin, F-75006 Paris, France; 10 CNRS UMR8104, F-75006 Paris, France; 11 Department of Endocrinology, Assistance Publique-Hôpitaux de Paris, Hôpital Cochin, F-75006 Paris, France; 12 Rare Adrenal Cancer Network COMETE, F-75006 Paris, France; 13 Department of Surgery, Assistance Publique-Hôpitaux de Paris, Hôpital Européen Georges Pompidou, F-75015 Paris, France

## Abstract

Pheochromocytomas and paragangliomas (PCCs/PGLs) are neural crest-derived tumours with a very strong genetic component. Here we report the first integrated genomic examination of a large collection of PCC/PGL. SNP array analysis reveals distinct copy-number patterns associated with genetic background. Whole-exome sequencing shows a low mutation rate of 0.3 mutations per megabase, with few recurrent somatic mutations in genes not previously associated with PCC/PGL. DNA methylation arrays and miRNA sequencing identify DNA methylation changes and miRNA expression clusters strongly associated with messenger RNA expression profiling. Overexpression of the miRNA cluster 182/96/183 is specific in *SDHB*-mutated tumours and induces malignant traits, whereas silencing of the imprinted *DLK1-MEG3* miRNA cluster appears as a potential driver in a subgroup of sporadic tumours. Altogether, the complete genomic landscape of PCC/PGL is mainly driven by distinct germline and/or somatic mutations in susceptibility genes and reveals different molecular entities, characterized by a set of unique genomic alterations.

## Abstract (web summary)

Pheochromocytomas and paragangliomas (PCCs/PGLs) are rare neuroendocrine tumours with a significant genetic component. Here, the authors carry out a multi-omic integrative characterization of PCC/PGL and reveal potential genomic alterations and regulatory mechanisms involved in the disease.

## Graphical abstract

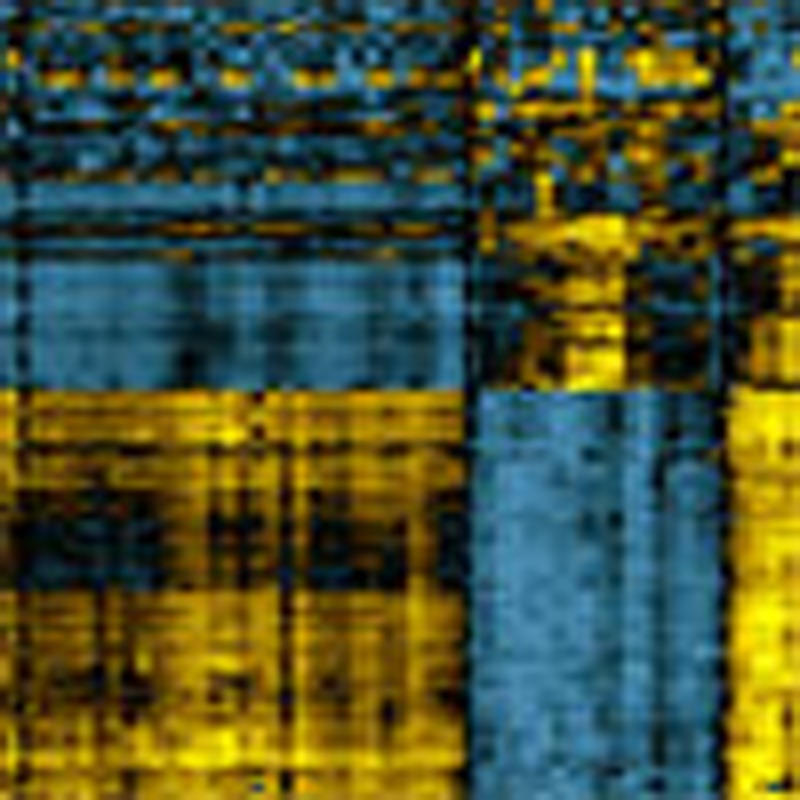

Pheochromocytomas (PCCs) and paragangliomas (PGLs) are rare neuroendocrine tumours derived from chromaffin cells of the adrenal medulla and paraganglia of the autonomic nervous system, respectively. Most PCCs/PGLs are benign, but 10% of PCCs and up to 40% of PGLs become malignant with a 5-year survival rate below 50%. The malignant form of the disease remains difficult to predict and cure, as there are neither established histopathological criteria to assess the risk of progression nor effective treatment. Compared with other cancers, PCCs/PGLs are characterized by a remarkable genetic determinism^1^. To date, at least 12 susceptibility genes have been identified comprising two oncogenes (*RET* and *HIF2A*) and ten tumour suppressors (*NF1*, *VHL*, *SDHA*, *SDHB*, *SDHC*, *SDHD*, *SDHAF2*, *FH*, *TMEM127* and *MAX*). Up to 40% of affected patients carry a germline mutation in one of these genes^1^. Germline mutations in either *SDHB* or *FH* have been associated with an increased risk of metastasis^2^,^3^,^4^. On the other hand, loss-of-function mutations in *NF1* and *VHL*, as well as activating mutations in *RET*, *HIF2A* and *HRAS*, have been reported at the somatic level in about 30% of these tumours^5^,^6^,^7^,^8^,^9^.

Transcriptome analyses identified expression clusters strongly correlated with known drivers^10^,^11^,^12^. Specifically, Clusters C1A and C1B, comprising *SDHx*- and *VHL*-mutated tumours, respectively, display a pseudohypoxic signature, whereas Cluster C2A, corresponding to *RET*-, *NF1*- and *TMEM127*-mutated tumours, displays activation of the RAS/mitogen-activated protein kinase (MAPK) signaling pathway. Clusters C2B and C2C are enriched in sporadic tumours. In addition, recent characterization of the methylation profile revealed that *SDHx* tumours display a hypermethylator phenotype^13^. Deregulated expression of microRNAs (miRNAs) and copy-number alterations (CNAs) have also been described^14^,^15^,^16^,^17^,^18^,^19^,^20^,^21^. However, to date, there is no study attempting to integrate multi-dimensional molecular data, which is necessary for a comprehensive understanding of the crucial mechanisms driving PCC/PGL tumorigenesis.

Here we present an integrated genomic analysis including whole-exome sequencing, single-nucleotide polymorphism (SNP) array, as well as mRNA, miRNA and DNA methylation profiling, aiming to characterize the major genomic alterations underlying PCCs/PGLs.

## Results

### Description and processing of the PCC/PGL COMETE cohort

A collection of 202 PCC/PGL samples from the French ‘Cortico et Médullosurrénale: les Tumeurs Endocrines’ (COMETE) Network was profiled on a set of genomic platforms. Tumour samples were collected from 190 patients, including 116 women and 74 men with a mean age at diagnosis of 42.4 years (range: 7–82 years; Supplementary Data 1). Overall, 168 (83.2%) tumours were PCCs, 25 (12.4%) were abdominal PGLs, 3 (1.5%) were thoracic PGLs and 6 (3%) were metastases. Twenty-seven (13.4%) tumours were from patients with a metastatic form of the disease. Importantly, all cases were previously genotyped for the presence or the absence of germline and somatic mutations in well-known susceptibility genes. For this study, we also screened the whole cohort for hotspot mutations of *HRAS* and found 10 of 202 (4.95%) mutated cases, corresponding exclusively to sporadic tumours (Supplementary Data 1).

SNP array (150 tumours), exome sequencing (31 tumour-normal pairs) and miRNA sequencing data (172 tumours) were generated specifically for this study, whereas DNA methylation (145 tumours) and mRNA expression data (188 tumours) were partially exploited in previous reports^5^,^6^,^13^,^22^. The collective molecular status derived from all of these platforms, excluding exome sequencing, was assigned to 128 tumours (Supplementary Fig. 1 and Supplementary Data 1).

### Somatic CNAs

**Figure 1:**
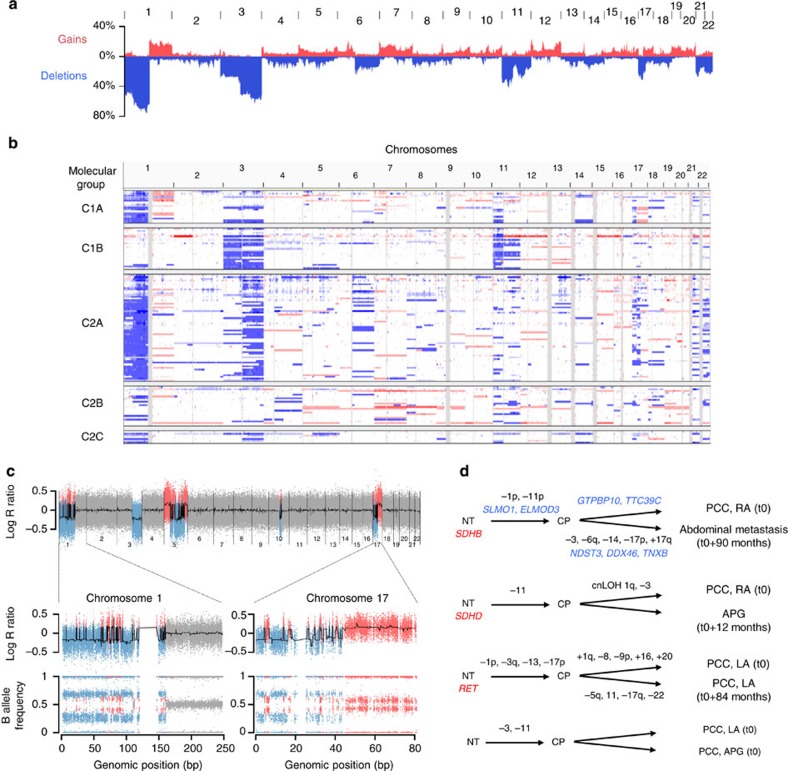
Copy-number alterations in PCC/PGL. (**a**) Pan-genomic frequency of gains (in red) and deletions (in blue) among the complete set of 150 PCC/PGL. (**b**) Overview of the copy-number profiles exported from the Integrative Genomics Viewer. Each line represents the profile of a tumour, with gains in red and deletions in blue. Samples are ordered according to the mRNA expression cluster they belong to, as indicated on the left. (**c**) Copy-number profile of a tumour presenting chromothripsis on chromosomes 1, 5 and 17. Top: Pan-genomic log R ratio profile. Bottom: log R ratio and B allele frequency profiles of chromosomes 1 and 17. These chromosomes display numerous breakpoints and a copy number oscillating between two values, classical of chromothripsis events. (**d**) Tumour progression trees reconstructed for four patients with primary tumour and co-occurring tumour/relapse/metastasis data. Tumours of the first patient were also analysed by exome sequencing. APG, abdominal paraganglioma; cnLOH, copy neutral loss of heterozygosity; CP, common precursor; LA, left adrenal; NT, normal tissue; PCC, pheochromocytoma; RA, right adrenal. In red, germline mutations; in blue, somatic mutations.

We first profiled somatic CNAs using SNP arrays. Overall, the degree of genome instability was low as determined by the fraction of aberrant arm score (median=15%). The most frequent CNAs were broad deletions involving chromosome arms 1p, 3p, 3q, 11p, 11q, 17p, 21 and 22 (Fig. 1a). Chromosome gains were less frequent, mostly affecting chromosome arm 1q and chromosome 7. Strikingly, tumours belonging to the different molecular groups displayed characteristic CNA patterns (Fig. 1b). Specifically, *SDHx* tumours (Cluster C1A) displayed a significant enrichment of 1q gains (*q*-value <0.05, Supplementary Table 1). Loss of chromosomal arm 1p encompassing the *SDHB* locus, as well as losses of 11p—mostly present in *SDHD* tumours—and 17p were also frequent, yet not exclusive of *SDHx* tumours. *VHL* tumours (Cluster C1B) were characterized by a combined deletion of chromosome 3 (harbouring *VHL*) and arm 11p. *RET*, *NF1* and sporadic tumours (Cluster C2A) displayed a high frequency of 1q and 3q deletions, and a significant enrichment of 17q11 deletions (*NF1* locus). Deletions of arm 6q and chromosomes 21 and 22 were also frequent in this group. Sporadic tumours included in Cluster C2B were enriched in gains of chromosome 7. A copy-neutral loss of heterozygosity (LOH) at 14q32 was significantly associated with Clusters C2B and C2C.

Although PCC/PGL present a fairly low degree of chromosome instability, three tumours (37, 49 and 188) presented evidence of chromothripsis, consistent with the proportion of 2–3% observed in most cancers^23^. Notably, chromosome arms 1p and 17p were strongly rearranged in the three cases (Fig. 1c), suggesting chromosome shattering may lead to the loss or gain of important drivers in these regions.

For four patients (18, 25, 42 and 80), we obtained the SNP array profiles of two tumours: coexisting tumours or the primary tumour, and a relapse or metastasis. We also generated exome data for one tumour/metastasis pair. Using the TuMult algorithm^24^, we reconstructed the order of copy-number changes in these tumours. We identified common alterations in every patient, confirming the clonal origin of their tumours. We could also distinguish early events, often comprising the loss of the wild-type copy of predisposing genes when a germline mutation is present, from late events that may be involved in tumour progression, including acquired somatic mutations and CNAs (Fig. 1d).

### Somatic mutations

**Figure 2:**
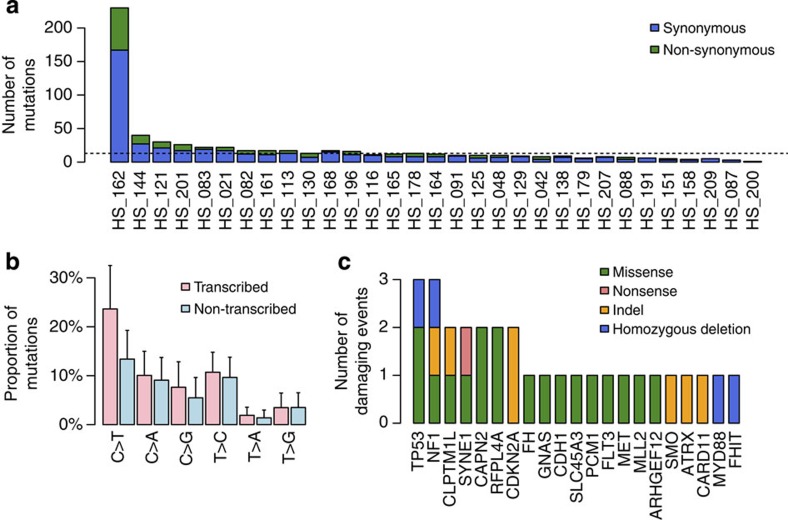
Whole-exome sequencing analysis of 31 PCC/PGL. (**a**) Number of synonymous and non-synonymous somatic mutations in the 31 PCC/PGL samples. (**b**) Relative proportions of the 6 possible base-pair substitutions on the transcribed and non-transcribed strands among the 616 point mutations identified in the exome sequences of 31 PCC/PGL. Error bars denote the s.d. (**c**) Number and type of damaging events for 7 genes altered in at least 2 cases, and 14 genes belonging to the Cancer Gene Census and altered in a single tumour.

To gain insights from the mutational landscape of PCCs/PGLs, we performed whole-exome analysis of 30 tumour-normal DNA pairs and one trio including the primary tumour and a metastasis. We enriched the exome series in tumours belonging to the C1A expression cluster (*n*=17) and sporadic tumours included in the C2A (*n*=3) and C2B (*n*=11) clusters, aiming to establish comparisons between these tumour groups (Supplementary Data 1). Among them, 15 were *SDHx*-related tumours; 1 tumour, belonging to the Cluster C1A, carried 1 germline and 1 somatic mutation in the *FH* gene as previously reported^13^; 2 sporadic tumours from the C2A cluster displayed somatic mutations in *NF1*; 13 were sporadic tumours with no mutation in known predisposing genes. We sequenced 333,765 exons from 20,975 genes to a mean coverage of 80-fold (Supplementary Fig. 2). Mutations were considered as somatic if they were found only in the tumour sample without evidence in the germline data. We identified a total of 672 somatic variants in coding regions or exon/intron boundaries: 616 single-nucleotide variants (SNVs) and 56 indels (Supplementary Data 2). PCCs/PGLs harboured a median of 3 synonymous and 9 non-synonymous mutations (range: 1–41 total mutations) (Fig. 2a), corresponding to a relatively low mutation rate of 0.3 mutations per megabase. The mutation rate did not differ between molecular subgroups or regarding the malignant status. The spectrum of mutations was enriched in C>T transitions (Fig. 2b), which are typically found in the majority of cancers analysed so far^25^. This result is compatible with spontaneous deamination rather than with a carcinogen-induced process. These mutations were also more frequent, yet not significant, in the transcribed strand.

We then combined damaging mutations (indels, nonsense or missense mutations predicted to be damaging by PolyPhen-2 software) and homozygous deletions obtained by SNP array (Supplementary Data 4), to identify putative driver genes in PCCs/PGLs. Seven genes presented at least two damaging events (Fig. 2c). As expected, we confirmed the *FH* and *NF1* mutations in corresponding tumours. Two other common cancer genes, *TP53* and *CDKN2A*, were altered in three (10%) and two (7%) cases, respectively, whereas the genes *CLPTM1L*, *SYNE1*, *CAPN2* and *RFPL4A* have not been yet implicated in cancer. Other genes of interest carrying damaging variants and listed in the Cancer Gene Census included *MET* (activating mutation p.E355K), *CDH1*, *ARHGEF12*, *SLC45A3*, *SMO*, *CARD11*, *MLL2*, *PCM1*, *ATRX*, *GNAS*, *FLT3* and *FHIT*. These genes were altered in a single tumour (Fig. 2c). Of note, the sample with the highest number of mutations (162) carries one somatic mutation in *POLE* and one in *POLD1*. All of these mutations were confirmed by Sanger sequencing (Supplementary Data 2). As expected, given the low mutation load of PCCs/PGLs, none of these variants was significantly mutated when MutSigCV was applied. To validate the exome-sequencing results, we screened the whole PCC/PGL cohort for mutations in *TP53*, *CDKN2A* and *MET* genes. In total, five (2.5%) tumours harbour hotspot mutations in *MET*, whereas no additional damaging variants were identified in *TP53* or *CDKN2A* genes (Supplementary Data 3).

### DNA methylation profiling

We previously identified three homogeneous DNA methylation-based subgroups of PCCs/PGLs: the M1 group corresponding to *SDHx*- and *FH*-mutated cases and displaying a hypermethylator phenotype, the M2 group comprising exclusively *VHL*-mutated samples and the M3 group comprising mostly tumours of the C2 expression cluster. We have already extensively described the DNA methylation changes occurring in the M1 cluster^13^. Here we extended our analyses to DNA methylation changes present in the two other groups. We identified 88 genes significantly hypermethylated in the M2 cluster (*VHL* related). Of these genes, 84 (95%) were also hypermethylated in the M1 cluster (Supplementary Data 5). However, these changes had little impact on gene expression, as only two genes (*PNMT* and *NMNAT3*) were both hypermethylated and downregulated in M2 tumours. We found no significant CpG island hypermethylation in tumours belonging to cluster M3. In contrast, these tumours displayed a widespread hypomethylation outside CpG islands (Supplementary Fig. 3a). Hypomethylation of the same loci was also encountered in M2 tumours but with lower amplitude (Supplementary Fig. 3b). DNA hypomethylation outside CpG islands may thus result from a similar mechanism operating with different intensities in the two subgroups.

### miRNA profiling

**Figure 3:**
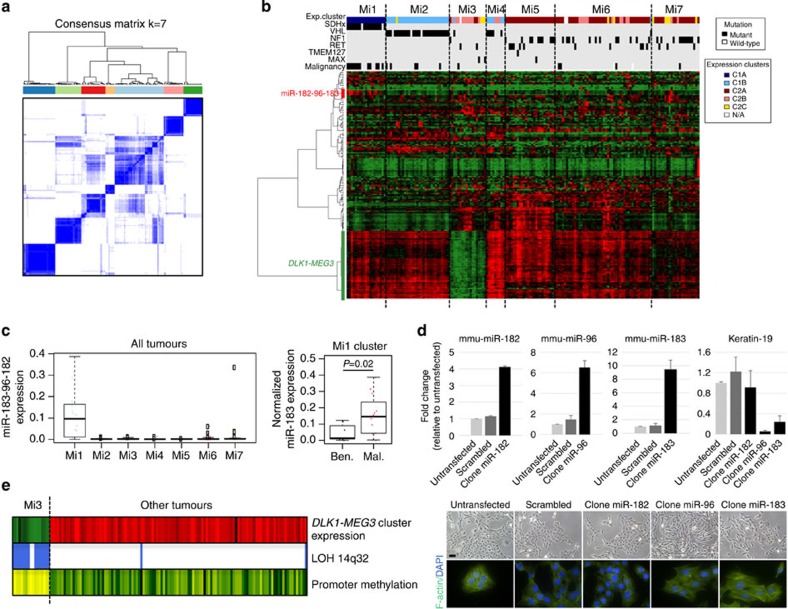
miRNA profiling of 172 PCC/PGL. (**a**) Unsupervised classification of 172 PCC/PGL identifies 7 stable miRNA expression clusters. The consensus matrix represents the similarity between samples. Consensus index values range from 0 (highly dissimilar profiles, white) to 1 (highly similar profiles, dark blue). Samples are ordered on the *x* and *y* axes according to consensus clustering, which is depicted above the heat map. (**b**) Heat map of miRNA expression profiles. The expression levels of the miRNAs with the most variant expression are shown by colour (red for high expression and green for low expression). Samples are ordered by miRNA expression subgroup, with mRNA expression subgroup and mutations in known driver genes depicted above the heatmap. (**c**) Box-and-whisker plots show the distribution of miR-183-96-182 expression levels relative to each tumour subgroup (left), and of miR-183 relative to malignancy among Mi1 tumours (right). Middle bar, median; box, interquartile range; bars extend to 1.5 times the interquartile range. Ben., benign; Mal., malignant. Error bars denote the s.d. (**d**) Overexpression of miRNAs 96/183 in mouse chromaffin cells induces an EMT-like phenotype. Histograms show the relative expression of mature miRs and keratin-19 (used as EMT marker) detected by quantitative reverse transcription–PCR in clones obtained after stable transfections with plasmids containing either scrambled or miRNA sequences. Error bars denote the s.e.m. The bottom panel shows the corresponding morphology changes as determined by F-actin immunostaining. Scale bar, 20 μm. (**e**) Deregulation of the *DLK1*-*MEG3* cluster in PCC/PGL of the Mi3 group. The mean expression levels of miRNAs belonging to the *DLK1*-*MEG3* cluster are indicated, together with the presence of a LOH at the locus (blue, LOH; white, no LOH) and the methylation levels at the promoter CpG island (yellow, fully methylated; green, hemimethylated; dark green, unmethylated).

To further characterize gene expression patterns of PCC/PGL tumours, we assayed miRNA expression levels in our cohort by Illumina sequencing. Unsupervised classification using the miRNome data of 172 PCCs/PGLs revealed 7 homogeneous subgroups (Mi1 to Mi7, Fig. 3a) strongly associated with mRNA expression subgroups (*P*=1.8e−63, *χ*^2^-test) and mutations in known driver genes (*P*=6.1e−39, *χ*^2^-test, Fig. 3b). Clusters Mi1 and Mi2 were more closely associated and corresponded to mRNA expression Cluster C1, whereas clusters Mi3 to Mi7 corresponded to mRNA expression Cluster C2. Clusters Mi1 and Mi2 comprised *SDHx*- and *VHL*-mutated tumours, respectively, whereas cluster Mi3 was enriched in sporadic tumours belonging to the C2B mRNA expression cluster. We used the DESeq package^26^ to identify differentially expressed miRNAs between the subgroups (Supplementary Data 6).

We found a significant upregulation of the miRNA cluster 182/96/183 in Mi1 (*SDHx*-related) tumours (Fig. 3c). We validated this finding using quantitative reverse transcription–PCR and confirmed that the highest expression is displayed by *SDHB*-mutated tumours (Supplementary Fig. 4). Of these, the miR-183 was significantly expressed in malignant cases (Fig. 3c). We demonstrated that ectopic expression of miR-183/96 but not miR-182 in immortalized mouse chromaffin cells (imCCs) induces an epithelial-to-mesenchymal transition (EMT) phenotype (Fig. 3d and Supplementary Fig. 5).

The Mi3 cluster, containing a subgroup of C2B, was characterized by a strong silencing of the imprinted *DLK1-MEG3* cluster (Fig. 3b,e). This genomic region harbours the largest cluster of miRNAs in the human genome^27^. Strikingly, 15 of 17 tumours belonging to cluster Mi3 displayed a LOH at the 14q32 locus harbouring *DLK1-MEG3*, compared with only 2 of 114 remaining cases (*P*=9.4e−16, Fisher’s exact test). We hypothesized that the loss of the maternal unmethylated allele may explain the repression of this imprinted miRNA cluster, and we indeed identified a shift in *MEG3* promoter methylation from hemimethylation to full methylation, specifically in Mi3 cases (*P*=4.1e−11, Wilcoxon rank-sum test, Fig. 3e).

### Multi-omics integration

**Figure 4:**
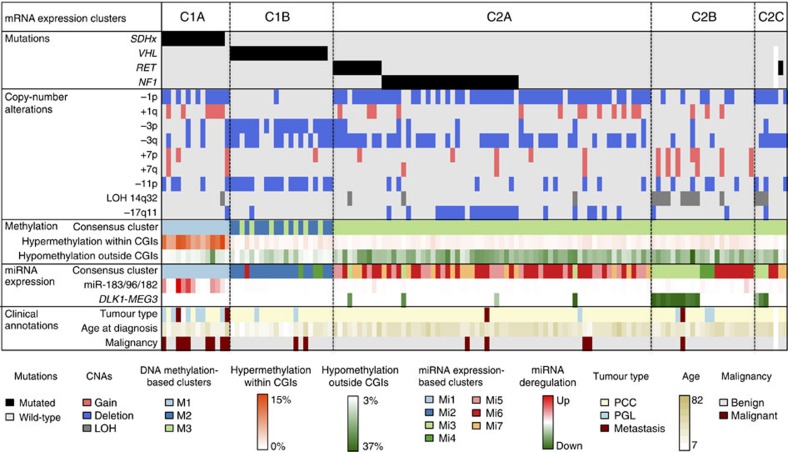
Integrative genomic characterization of PCC/PGL. The main genetic, epigenetic and transcriptional changes affecting PCC/PGL are represented for the 128 samples analysed by SNP, DNA methylation, mRNA expression arrays, miRNA sequencing and targeted sequencing of known driver genes. Samples are ordered according to the mRNA cluster they belong to. Clinical features associated with molecular groups are also depicted at the bottom.

The combination of the main findings obtained with each omics data set defines the molecular portrait of each PCC/PGL subgroup (Fig. 4). C1A tumours are driven by germline mutations affecting *SDHx* genes. These tumours harbour frequent deletions of 1p and gains of 1q, display a hypermethylator phenotype and an overexpression of the miRNA cluster 182/96/183. This subgroup mostly comprises PGLs and malignant cases. C1B tumours are driven by *VHL* mutations. These tumours display a co-deletion of chromosome 3 and chromosome arm 11p, promoter hypermethylation of a few targets and a low-amplitude hypomethylation outside CpG islands. C2A tumours are mainly driven by *RET*, *NF1* and *HRAS* mutations. They harbour frequent 1p and 3q deletions, as well as 17q11 deletions in *NF1*-related cases. This group displays a strong hypomethylation outside CpG islands, such as the C2B and C2C groups. C2B tumours have no recurrent mutations but display frequent gains of chromosome 7, as well as a downregulation of the tumour suppressor *DLK1-MEG3* miRNA cluster following LOH at the 14q32 locus, also encountered in the C2C group. C2C tumours have only few CNAs, including deletions of 1p and 3q, but no chromosomal gains. Overall, the C2 group comprises mainly benign PCCs with a later age of onset.

## Discussion

The integrative genomic analysis of the well-annotated and genotyped COMETE cohort demonstrated that mutation status in PCC/PGL susceptibility genes is strongly correlated with multi-omics data. The somatic inactivation of the normal allele in PCC/PGL tumour suppressors such as *SDHx*, *VHL* and *NF1* tumours is driven by large deletions at the corresponding loci, but not by somatic mutations or epigenetic modifications. Given that these alterations involve large genomic segments, it remains challenging to identify other potential drivers and we cannot formally exclude the possibility that a concomitant loss of another tumour suppressor participates to tumorigenesis. Nevertheless, loss of 1p (*SDHB* locus) is broadly observed in PCC/PGL and was also encountered in cases displaying chromothripsis.

Regarding the mutational landscape, PCCs/PGLs are similar to other neural crest-derived tumours. Although they display a low mutation rate similar to neuroblastomas, medulloblastomas and ependymomas^28^,^29^,^30^,^31^, PCCs/PGLs have mutations in *TP53*, *CDKN2A* and the RAS/MAPK signalling pathway that have also been found in melanomas and glioblastomas^32^,^33^.

Importantly, we did not find any recurrent mutations in PCCs/PGLs, but it is expected that analysis of more samples could help identify significant genes of low mutation frequency^34^. In fact, our validation screening revealed frequent mutations in *MET*, particularly in C2A and C2B tumours. This gene encodes for a receptor with tyrosine kinase activity that triggers the RAS/MAPK signalling pathway frequently altered in PCCs/PGLs and therefore can be considered as a potential driver and theranostic biomarker^35^. We also point out mutations in *ATRX* and *MLL2*, two chromatin-remodelling genes, as they are frequently altered in gliomas, neuroblastomas, medulloblastomas and neuroendocrine tumours^28^,^29^,^30^,^36^,^37^,^38^,^39^,^40^ and may therefore constitute a converging tumorigenic pathway for these related tumour types.

We previously found that *SDHx* and *FH* mutations establish a hypermethylator phenotype in PGLs^4^,^13^. In this regard, it is worth mentioning that similar results have been observed for at least two other neural crest-derived tumours, ependymomas and *IDH*-dependent glioblastomas^31^,^33^, as well as in gastro-intestinal stromal tumours^41^. Increased CpG methylation induced by accumulation of metabolic intermediates (oncometabolites) reflects a block of the differentiation programme, thus probably promoting an increase in the number of stem and progenitor cells. Epigenetic modifiers would be a pertinent therapeutic strategy for this type of malignancy. On the other hand, the significance of the prevalent hypomethylation outside CpG islands found in Cluster 2 PCCs/PGLs remains unclear. Indeed, hypomethylation has classically been associated with genomic instability. In this subgroup of tumours, we have however not observed an increase in chromosome instability, and the pathophysiological consequences of this phenotype are still to be further investigated. In addition, we show here that *PNMT* gene encoding for the enzyme catalysing the conversion of noradrenaline to adrenaline is hypermethylated and downregulated in *VHL* tumours, suggesting that epigenetic silencing of this gene explains the noradrenergic phenotype of both *VHL*- and *SDHx*-mutated tumours^42^.

Regarding miRNA expression changes, a high expression of miR-96 and miR-183 in *SDHB*-mutated tumours has been previously observed^17^. Here we extended this observation to the whole miRNA cluster miR-182/96/183 and further verified that this overexpression is not related with CNA at this miR locus. Of these miRNAs, it seems that miR-183 is specific for malignant tumours, suggesting a potential prognostic value^16^. We previously reported that *SDHB*-mutated tumours display the activation of the EMT programme^43^. Here we further demonstrated that overexpression of miR-183 or miR-96 in mouse chromaffin cells induces an EMT-like phenotype. This miRNA cluster is upregulated in other invasive cancers including neural crest-derived tumours such as medulloblastomas, gliomas and melanomas^44^,^45^,^46^,^47^,^48^. Moreover, these miRs are highly expressed in neural stem cells and downregulated during differentiation^49^. Overexpression of miR-183 or miR-96 in PC12 cells blocks Nerve growth factor (NGF)-mediated differentiation^17^. Altogether, these observations suggest that these miRNAs may contribute to the maintenance of the undifferentiated state of *SDHB*-mutated tumours. The specific targets as well as the precise role of miR-182 await further functional characterization.

The miRNA profiling of PCC/PGL also uncovered a novel subgroup of sporadic tumours carrying a shutdown in the expression of the imprinted *DLK1-MEG3* cluster located in 14q32, an alteration previously associated with *MAX* tumours^17^. It is worth mentioning that this cluster is highly expressed in normal adrenal and other endocrine tissues^50^. The fact that both LOH and focal deletions of this genomic region were present, strongly suggests a tumour suppressor role of these miRNAs. Interestingly, in adrenocortical carcinoma, this alteration was also found in a benign subgroup without known driver mutation^51^. Therefore, downregulation of this cluster may be sufficient to trigger tumorigenesis in adrenal tissues.

In conclusion, this integrative genomic study provides evidence for strong concordance between multi-omics data and genetic status, thus demonstrating the crucial role of predisposing mutations as being the main drivers of PCCs/PGLs. This comprehensive analysis further illustrates the functional interdependence between genomic and epigenomic dysregulations. Ultimately, PCC/PGL subtypes can be defined by a set of unique genomic alterations and most probably represent different molecular entities. In the near future, omics-based tests should be developed to offer access to a precise molecular classification of PCCs/PGLs, to practicing clinicians. The knowledge of specific genomic alterations should provide a real help for individual patient management and should guide the choice of targeted therapy for malignant cases. Therefore, our findings pave the way towards omics-based clinical management and personalized medicine for patients with PCCs/PGLs.

## Methods

### Tumour samples

The tumour and blood samples were collected prospectively by the French COMETE Network. Ethical approval for the study was obtained from the institutional review board (Comité de Protection des Personnes Ile de France III, June 2012).

Written informed consent for the sample collection and subsequent analyses was obtained from all patients. The procedures used for PCC/PGL diagnosis were in accordance with both internal and international clinical practice guidelines^52^,^53^. Diagnosis was confirmed by histology in every case. A total of 202 consecutive cases of PCCs/PGLs, recruited over 15 years (1993–2008), were included in this study. Fresh tumour samples collected during surgery were immediately frozen and stored in liquid nitrogen until processing.

### Nucleic acids extraction

Germline DNA was extracted from leukocytes using standard protocols. Tumour samples (30–50 mg) were powdered under liquid nitrogen. DNA was extracted and purified using a QIAamp DNA Mini Kit or an Allprep Kit (Qiagen) and RNA was extracted using an RNeasy Mini Kit (Qiagen). miRNA was extracted using a miRNeasy Mini Kit (Qiagen). Concentration of nucleic acids was determined using a Nanodrop ND-1000 instrument (Nyxor Biotech). RNA quality was assessed by electrophoresis on a Bioanalyzer 2100 (Agilent Technologies). A 28S/18S ratio above 1.5 was considered to rule out degradation.

### Genetic testing and validation of somatic mutations

Mutation analysis of PCC/PGL susceptibility genes was performed as previously described^4^,^5^,^6^. Screening of *CDKN2A* gene and hotspot mutations in *HRAS* (exons 2, 3, 4 and 5), *TP53* (exons 4, 5, 6, 7, 8, 9, 10 and 11) and *MET* (exons 2, 14, 16 and 19) genes in the whole cohort, as well as validation of 20 mutations identified by exome sequencing in cancer genes (Supplementary Data 2) were performed by Sanger sequencing. The somatic nature of mutations was confirmed by sequencing tumour DNA and their paired blood DNA. The primer sequences and PCR conditions are available on request.

### Analysis of copy-number data

DNA samples from 150 PCC/PGL (145 primary tumours and 5 relapses) were analysed with Illumina HumanCNV610-Quad v1.0. Hybridization was carried out by Integragen SA (Evry, France), following the manufacturer’s recommendations. Raw fluorescent signals were launched into BeadStudio software (Illumina) and normalized^54^ to obtain log R ratio and B allele frequency values. Genomic profiles were divided into homogeneous segments by applying the circular binary segmentation algorithm^55^ to both log R ratio and B allele frequency values. The ploidy of each sample, the level of contamination with normal cells and the allele-specific copy number of each segment was determined using the Genome Alteration Print method^56^. The overall genomic instability of each sample was quantified as the fraction of aberrant arm score^57^. When several samples were available from the same patient, tumour progression trees were reconstructed using the TuMult algorithm^24^.

### Sequencing data processing and mutation detection

Whole-exome sequencing was performed in 30 pairs of PCCs/PGLs and matched normal samples (from blood), and 1 trio with a primary tumour and metastasis. In all tumour samples, the ratio of tumour cells to non-tumour cells was determined to be >50% and this value was confirmed by SNP array analysis. Sequence capture and exome sequencing were performed by Integragen as described elsewhere^57^. Briefly, Agilent in-solution enrichment (SureSelect Human All Exon Kit v4+UTR) with the provided biotinylated oligonucleotide probe library (Human All Exon v4+UTR–70 Mb) was used for DNA sequence capture, enrichment and elution from the tumour and matched blood DNA samples. The eluted fraction was amplified by PCR and sequenced on an Illumina HiSeq 2000 sequencer as paired-end 75-bp reads. Image analysis and base calling were performed using Illumina Real-Time Analysis Pipeline version 1.12.4.2 with default parameters. Illumina CASAVA 1.8.2 software was used to align reads against the hg19 genome build (GRCh37) and to detect SNVs and indels. We used the ELANDv2 algorithm for alignment. Targeted regions were sequenced to an average depth of 80 × . We applied a previously described pipeline^51^, with some modifications, to generate a list of somatic variants located in coding regions, plus four intronic bases corresponding to consensus splice sites. Quality-control filtering removed variants sequenced in <10 reads, with <3 variant calls or with QPHRED of <10. Variants were considered to be of somatic origin when the frequency of variant reads was ≥10% in the tumour and <5% in the normal counterpart, with a significant enrichment of variant cells in the tumour as assessed through a Fisher’s exact test. Common polymorphisms (with reported frequency of >1%) were removed by comparison with dbSNP135, the 1000 Genomes Project database and a proprietary database of exomes from normal tissues. Missense variants were classified as ‘benign’, ‘possibly damaging’ or ‘probably damaging’ using PolyPhen-2 software (http://genetics.bwh.harvard.edu/pph2/). AlamutVisual Software version 2.4.6 (http://www.interactive-biosoftware.com/software.html) was used for nomenclature of gene variations. Somatic mutations occurring in at least two samples were validated visually using the Integrative Genomics Viewer^58^. The mean number of somatic mutations per sample (three silent and nine non-silent) comprised exonic SNVs and indels. The mutation rate in each tumour was evaluated by dividing the number of somatic mutations by the number of exonic bases covered by ≥10 × in both the tumour and normal samples. To identify mutations with transcriptional strand bias, we used a binomial test to assess whether the proportions of each type of somatic substitution occurring on the transcribed strand differed significantly from 0.5.

### miRNA profiling

miRNA expression was performed in 172 PCC/PGL. Multiplexed miRNA libraries were prepared using a PCR barcoding method^59^ and sequenced on an Illumina HiSeq 2000 sequencer. Image analysis, base calling, demultiplexing and conversion of BCL to FASTQ format were carried out using Illumina CASAVA 1.8.2 software. Adaptor sequences were removed using mirExpress software^60^ and FASTA files for each sample were processed by miRanalyzer0.3 software^61^, to quantify read counts for each miRNA referenced in mirBase74 v18. In total, 763 miRNAs were expressed (>10 reads) in at least 2 samples and were used for unsupervised classification. The miRNA count data were log2 transformed, divided by the total number of reads in each sample and centred on the mean expression level of each gene. Consensus clustering was then performed^62^ and the optimal number of clusters (*K*=7) was determined from cumulative distribution function (CDF) curves. We used the Bioconductor DESeq package^26^ to identify differentially expressed miRNAs. For validation of the expression of miRNA cluster 182/96/183 in tumour tissues and murine cell lines, we used the following TaqMan assays (Applied Biosystems): mmu-miR-183 (ID 002269), mmu-miR-96 (ID 000186), hsa-miR-182 (ID 002334) and mmu-miR-182 (ID 002599), hsa-miR-324-3p (ID 002161), has-miR-320 (ID 002277) and U6 snRNA (ID 001973). Briefly, 10 ng of RNA previously treated with Turbo DNase was reverse transcribed. Amplifications were performed in a StepOne Plus Real-Time PCR system. The expression of U6 or the mean expression of miR-320 and miR-324 were used as normalization control in murine cells and tumours, respectively.

### Gene expression data

Gene expression profiles were available from a previous study, in which we identified five stable PGL/PCC subgroups, significantly associated with clinical features and gene mutations^6^. HG-U133 Plus 2.0 Affymetrix GeneChip data from this previous study are available online as ArrayExpress entry E-MTAB-733 (http://www.ebi.ac.uk/arrayexpress/).

### DNA methylation arrays

Whole-genome DNA methylation was analysed in 145 tumour samples using the Illumina Infinium HumanMethylation27 assay as previously reported^13^. Data from this previous study are accessible through the NCBI’s Gene Expression Omnibus series accession number GSE43298 (http://www.ncbi.nlm.nih.gov/geo).

### Cellular assays

imCCs^13^ were cultured in medium DMEM+Glutamax (GIBCO) supplemented with 10% fetal bovine serum (GIBCO) and maintained in a humidified 5% CO_2_ atmosphere at 37 °C. For stable transfections of miRs, we used the Origen plasmid mmu-miR-183 (catalogue number SC400806), which also contains the mmu-miR-96 sequence, whereas both scrambled and mmu-miR-182 sequences were cloned in pCMV6-A-Puro Vectors. MegaTran 1.0 was used as transfection reagent (CliniSciences). Clones expressing one of the miRs belonging to the cluster 182/96/183 were obtained by limited dilution assays.

For immunofluorescence, microscope slides (Thermo scientific) were coated with Poly-D-lysine (100 μg ml^−1^) for 3 h at 37 °C and washed with PBS 1 × without calcium. Cells seeded for 24 h were assayed for F-actin staining. Briefly, cells were fixed in 3% formaldehyde and 2% sucrose for 20 min at room temperature. Next, cells were washed twice with PBS 1 × and permeabilized for 10 min. A stock solution of phalloidin–tetramethylrhodamine isothiocyanate (Sigma) in dimethyl sulfoxide at 0.5 mg ml^−1^ was used at a concentration of 1:1,000 in PBS 1 × for 1 h at room temperature. Cells were washed several times to remove the conjugate and stained with 0.5 mg ml^−1^ 4′,6-diamidino-2-phenylindole.

Cell migration capacity of transfected imCC was assessed by performing *in vitro* scratch assays. Closure of the wounds was measured after 12 h^13^. For invasion assays, 75,000 cells per well (24-well format plates) were seeded on cell wall filters (8.0 μm pore size) containing a layer of growth factor reduced matrigel (BD Biosciences) diluted 1:4 in serum-free culture media. Cells were allowed to traverse the Matrigel for a period of 40 h after which they were fixed with methanol 15 min and then stained with toluidine blue 1% for 30 min. Cells were removed from the upper side of the chamber with cotton swabs. Images were taken from two independent experiments for quantifications.

## Additional information

**How to cite this article**: Castro-Vega, L. J. *et al*. Multi-omics analysis defines core genomic alterations in pheochromocytomas and paragangliomas. *Nat. Commun.* 6:6044 doi: 10.1038/ncomms7044 (2015).

**Accession codes**: Exome-sequencing data has been deposited in the European Genome-phenome Archive under the accession code EGAS00001000933. SNP array data has been deposited in ArrayExpress under the accession code E-MTAB-2817. miRNA sequencing data has been deposited in ArrayExpress under the accession code E-MTAB-2833.

## Supplementary Material

Supplementary Figures, Supplementary Table and Supplementary Data Supplementary Figure 1-5 and Supplementary Table 1

Supplementary Data 1 Clinical and genetic characteristics of analyzed PCC/PGL.

Supplementary Data 2 List of somatic variants identified by whole exome sequencing in a set of 31 PCC/PGL.

Supplementary Data 3 List of non-pathogenic and pathogenic variants in TP53, MET and CDKN2A genes identified in the PCC/PGL cohort.

Supplementary Data 4 List of homozygous deletions identified by SNP arrays.

Supplementary Data 5 List of genes differentially methylated/expressed in each group of tumors.

Supplementary Data 6 List of differentially expressed miRNAs in each group of tumors.
