# Supplementary material for: Multi-omics analysis defines core genomic alterations in pheochromocytomas and paragangliomas
Source: Nat Commun. 2015 Jan 27;6:6044. doi: 10.1038/ncomms7044 (PMC4354166; doi:10.1038/ncomms7044)
Supplement: Supplementary Figures, Supplementary Table and Supplementary Data — Supplementary Figure 1-5 and Supplementary Table 1 [file ncomms7044-s1.pdf]

Supplementary Figures

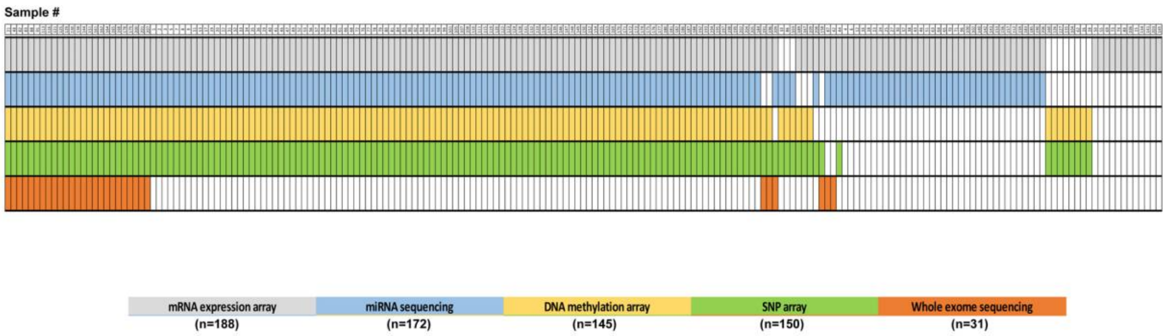

Supplementary Figure 1. Characterization of tumors using genomic platforms. The color code indicates the type of genomic analysis performed per sample.

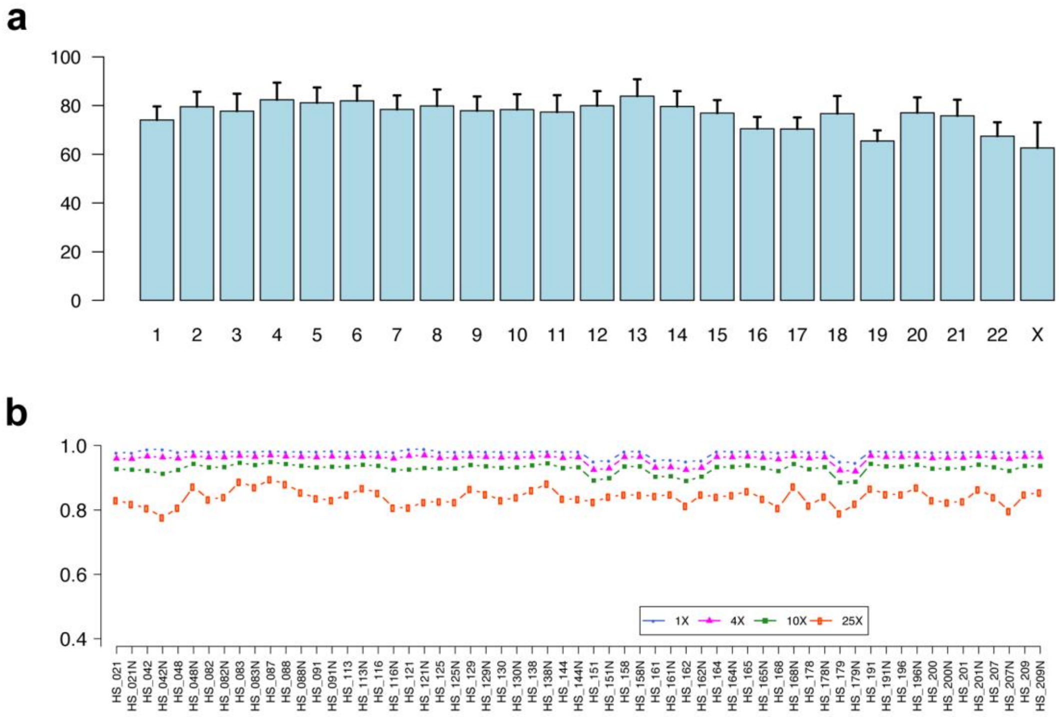

Supplementary Figure 2. Coverage of whole exome sequencing data. (a) Mean sequencing depth for each chromosome across the cohort. **(b)** Coverage at 1X, 4X, 10X and 25X for each sample.

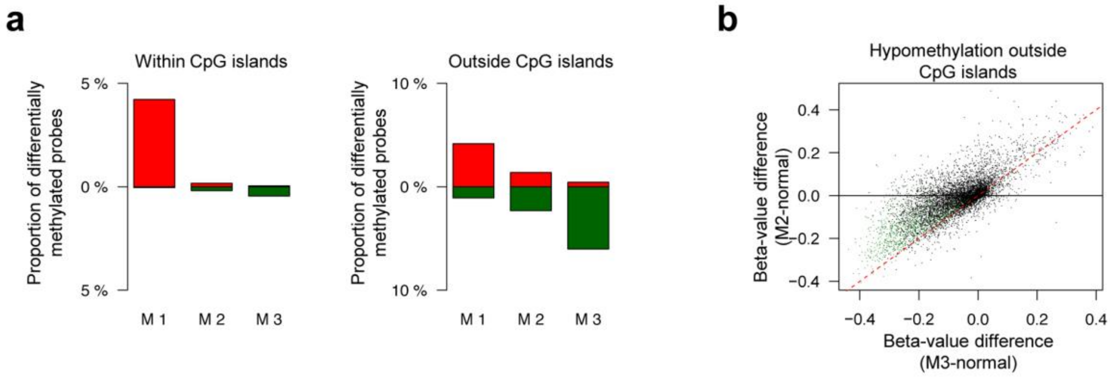

Supplementary Figure 3. DNA methylation changes in each tumor subgroup. (**a)** Proportion of probes differentially methylated (hypermethylated: red; hypomethylated: green) between each cluster of tumors and normal controls (*P* < 0.01, Wilcoxon rank sum test). **(b)** Correlation between DNA hypomethylation outside CpG islands in M2 and M3 tumors. Probes significantly hypomethylated in M3 tumors are colored green.

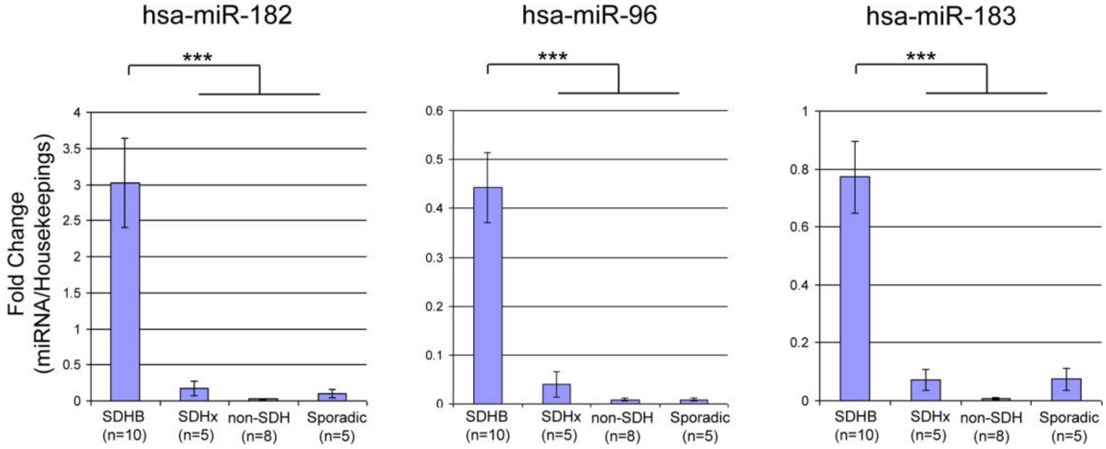

Supplementary Figure 4. Validation of miRNA cluster 182/96/183 expression in PCC/PGL. The histograms show the mean expression for each miRNA in each tumor type, relative to the mean expression of miR-320 and miR-324 used as housekeepings (normalization controls). Detection was performed using RT-qPCR with specific Taqman assays. The highest expression is displayed by *SDHB*-mutated tumors (***p-value <0.0001, two-tailed t test).

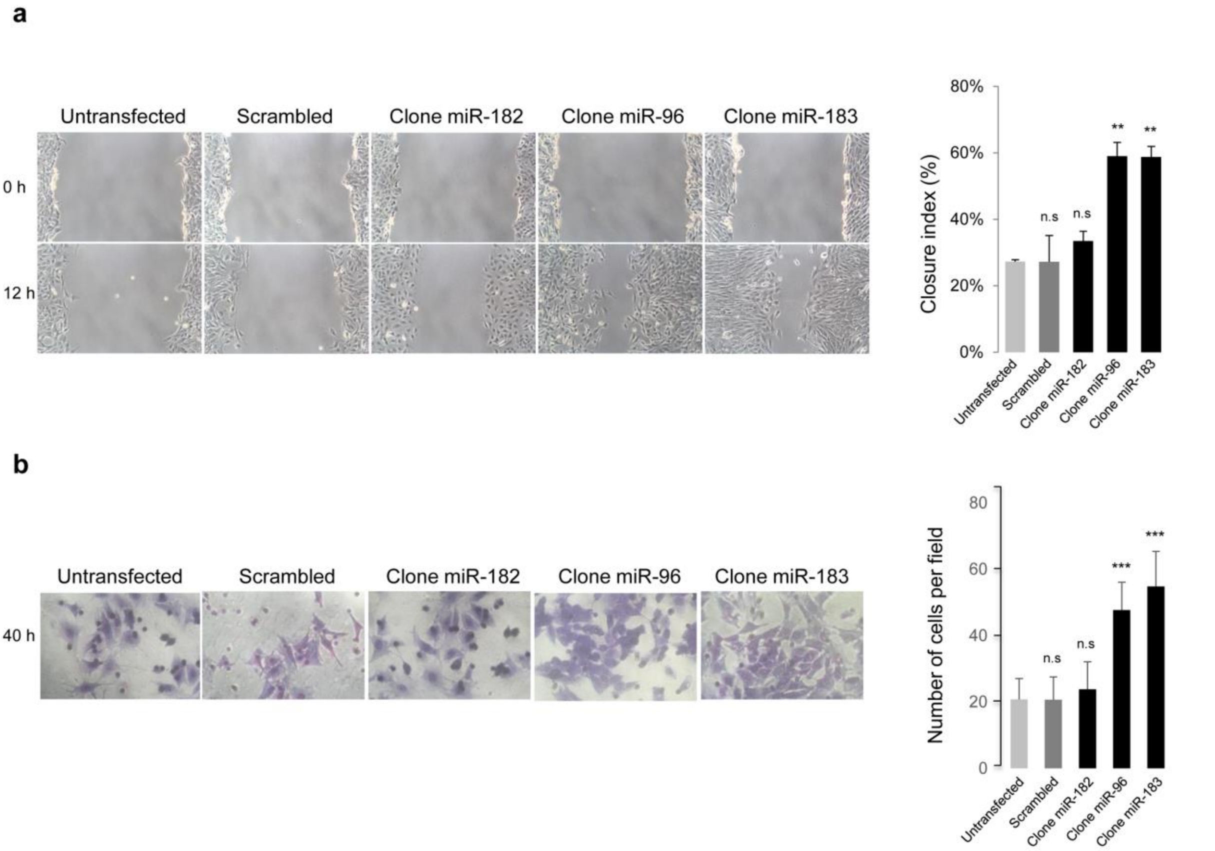

Supplementary Figure 5. Overexpression of miR-96 or 183 enhances the migratory and invasive capacity of imCC cells. (a) Scratch assay. Representative images (10X) of cells acquired at 0 h and 12 h. The histogram (on the right) shows the closure index (%) for each cell line quantified at 12 h. Error bars indicate standard deviation (n=10) (n.s: not significant; **p-value <0.01, two-tailed t tests compared to untransfected cells) **(b)** Invasion assay. Representative images (32X) of invaded cells on filters were acquired at 40 h. Error bars indicate standard deviation (n=10) (n.s: not significant; **p-value <0.001, two-tailed t tests compared to untransfected cells).

Supplementary Table

Aberration	Frequency in C1A tumors	Frequency in C1B tumors	Frequency in C2A tumors	Frequency in C2B tumors	Frequency in C2C tumors	q-value
-1p	68.8	9.1	80.6	27.3	85.7	1.13e-08
+1q	50	0	19.4	13.6	0	0.00386
-3p	18.8	86.4	13.4	4.5	14.3	5.37e-10
-3q	25	63.6	64.2	18.2	85.7	4.33e-04
+7p	25	4.5	7.5	36.4	0	0.00643
+7q	12.5	0	4.5	27.3	0	0.0114
-11p	31.2	72.7	13.4	27.3	42.9	3.56e-05
LOH 14q32	6.2	0	4.5	40.9	42.9	1.88e-05
-17q11	6.2	9.1	35.8	9.1	14.3	0.014

Supplementary Table 1. Copy number alterations associated with molecular clusters.
